# Shoe choice may affect fencing lunge attack performance

**DOI:** 10.3389/fbioe.2024.1276025

**Published:** 2024-02-14

**Authors:** San-Tsai Wang, Che-Chia Chang, Te Chao, Andrew Nicholls, Yung-Shen Tsai

**Affiliations:** ^1^ Office of Physical Education, Ming Chuan University, Taipei, Taiwan; ^2^ Graduate Institute of Sports Science, University of Taipei, Taipei, Taiwan; ^3^ Graduate Institute of Sports Equipment Technology, University of Taipei, Taipei, Taiwan

**Keywords:** attack time, heel cup, lower limbs, biomechanics, motion analysis

## Abstract

The purpose of this study was to investigate the differences in attack time and lower limb biomechanics when performing fencing lunge with fencing shoes (FS) and commonly used court shoes (CS). Additionally, the study aimed to evaluate whether fencing shoes with a heel cup (FSH) could reduce lower limb impact. Thirteen female collegiate fencers who had participated in national-level competitions were recruited for this study. Participants performed the lunge on a human-shaped target while wearing FS, FSH, or CS in a randomized order. Biomechanical data were collected using a 3D motion analysis system synchronized with a force plate. A signal light, and an accelerometer were attached to the target’s head to initiate lunge movement and detect hit moment for calculating attack time. Attack time was significantly shorter when wearing FS (0.92 ± 0.05 s) and FSH (0.93 ± 0.07 s) compared to CS (0.96 ± 0.06 s). The maximum angular velocity of ankle plantarflexion in rear foot push-off phase was significantly slower when wearing FS and FSH than when wearing CS. The maximum knee posterior shear force, maximum knee flexion moment, and maximum ankle medial shear force during the front foot step phase were significantly greater when wearing FS than when wearing CS. These forces were significantly reduced or nearly significantly reduced when wearing FSH, and there were no significant differences compared to wearing CS. The maximum ankle medial shear force during the push-off phase in rear foot was the greatest when wearing FS but decreased significantly when using FSH. However, this force was still greater than when wearing CS. Wearing FS resulted in a higher loading rate (LR) on the front foot. This LR was reduced when a heel cup was used but still remained higher than when wearing CS. There were no significant differences in the forward extension of body, maximum ground reaction force, or center of pressure displacement during front foot step and rear foot push-off phases among the three shoe conditions. Wearing FS can enhance lunge performance, and the use of a heel cup can effectively reduce lower limb impact.

## 1 Introduction

The popularity and competitiveness of fencing are rapidly increasing across various gender and age groups (Thompson et al., 2022). Fencing is an explosive, asymmetric sport that involves rapid movements. Athletes must constantly perform a series of advances, retreats, and impacts, with different roles for the front and rear legs (Geil, 2002; Chen et al., 2017). The asymmetric actions and momentum change rapidly during competition. Regardless of the type of sword used, the lunge is one of the main attacking actions in fencing, and it is a type of bow step action (Turner et al., 2014). When a fencer performs a lunge attack, their body weight transfers to the front supporting leg, and the body moves forward by pushing off with the rear leg. Elite fencers can generate greater horizontal velocity by swiftly shifting their center of gravity (COG) and extensively extending their bodies forward (Chen et al., 2017; Guan et al., 2018). Performing a lower position further enhances the adaptability and unpredictability of the attack for the opponent (Trautmann et al., 2011; Bottoms et al., 2013; Chen et al., 2017; Magnani & Defrasne Ait-Said, 2021). Previous studies have shown that improving the performance of the bow step in fencing requires increasing limb extension, joint angular velocity, and reducing reaction and movement time. Elite fencers have a reaction time of about 350 ms and a completion time of about 600 ms for attacks (di Cagno et al., 2020; Williams and Walmsley, 2000). In high-speed competition, shortening the fencer’s reaction and movement time is crucial, not only to increase the chances of a successful attack but also to reduce the chances of being counterattacked (Gholipour et al., 2008).

Fencers wear different types of shoes at various levels of fencing competitions, such as court shoes and fencing-specific shoes (Geil, 2002). Court shoes are chosen for their affordability in comparison to fencing shoes. These shoes share some common features, such as better grip and lighter weight. Fencing requires rapid movements that rely on instant power transmission, so fencing shoes need to be lightweight, with good grip and transmission capabilities, as well as smooth and responsive edges (Lin, 2020). While court shoes offer excellent grip, stability, cushioning, and traction control (Reinschmidt and Nigg, 2000; Geil, 2002), they may hinder the smoothness of the pushing and kicking movement required in fencing due to their tendency to have more shock-absorbing functions (Geil, 2002). Therefore, using court shoes may result in power loss and reduced fluidity of movement during fencing.

Previous studies have found that the peak force required for pushing off during the fencing action is about 14.61 N kg⁻^1^, while the horizontal reaction force is about 7 N kg⁻^1^ (Gutiérrez-Dávila et al., 2013; Guilhem et al., 2014; Turner et al., 2016). To enhance power transmission capabilities and prevent power absorption by the sole during the pushing-off stage, fencing shoes are purposefully designed, distinguishing them from running shoes or sneakers that prioritize shock absorption in the sole. Nevertheless, the functions of the front and rear legs vary, with the rear leg primarily involved in pushing, while the front leg necessitates forward extension and absorption to withstand impact (Geil, 2002). In instances where fencing shoes lack adequate shock absorption, there is a potential for excessive loading on the front foot during a lunge attack. Consequently, fencers commonly use a heel cup to mitigate the impact loading.

Fencing-specific shoes have been modified to accommodate the unique demands of the sport. However, Geil (2002) found that fencing shoes still exhibit greater plantar pressure on the forefoot during fencing offensive movements compared to court shoes. This difference may be amplified among athletes of different levels. In practical, higher-level fencers with better technical proficiency tend to exhibit smoother buffering actions from the heel to the entire plantar surface upon forefoot landing. Conversely, fencers with average technical proficiency and skill level often initiate the movement by forcefully stopping the forward momentum with their heels before fully engaging the entire plantar surface. It has been reported that elite fencers generate a higher knee joint moment and power during the lunge movement compared to intermediate-level fencers, attributed to a higher ground reaction force (GRF) (Guan et al., 2018). Additionally, elite fencers also demonstrated a faster horizontal peak velocity. The combination of a faster lunge attack and a higher GRF would result in greater stress on the lower limb joints. Therefore, some individuals in competitive settings use a heel cup in conjunction with fencing shoes to address this issue in practical. A heel cup functions as cushioning inserts, similar to insoles, but only covers the heel region (Perhamre et al., 2012). Coaches and athletes who utilize a heel cup believe that they provide greater impact reduction to the heel without impeding the transfer of propulsive forces from the arch and forefoot. Consequently, the selection of court shoes, fencing-specific shoes, and fencing shoes with a heel cup for competition and training becomes an important issue among athletes of different skill levels.

Although fencing shoes are one of the most used types of shoes in fencing competitions, there is still a lack of data to verify whether fencing shoes have any differences in sports performance compared to commonly used court shoes. It is also unclear whether the use of a heel cup can truly reduce the impact load on the heel of the athlete. Therefore, the purpose of this study was to investigate whether different types of shoes (court shoes and fencing shoes) will have an impact on the sports performance (attack time) and lower limb kinematics (knee and ankle joint angle and angular velocity) and kinetics (knee and ankle joint forces and moments, GRF, loading rate (LR), and center of pressure (COP) movement) parameters of the fencing lunge. At the same time, the study examined whether the use of a heel cup with fencing shoes can effectively change the lower limb kinetic parameters. The study hypothesis was that fencing shoes would have better sports performance compared to court shoes, and that the use of a heel cup with fencing shoes could more effectively reduce the load on lower limb joints than wearing fencing shoes alone.

## 2 Materials and methods

### 2.1 Participants

A convenience sampling approach was adopted for this study. This study recruited 13 female collegiate fencers (age: 20.4 ± 3.2 years; height: 166.6 ± 6.2 cm; weight: 56.5 ± 7.4 kg; training age: 6.0 ± 3.0 years) who had not suffered from any neuromusculoskeletal injuries that could affect sports performance within the past 6 months. Inclusion criteria were at least 2 years of fencing experience, training at least 3 days a week, and having participated in national-level competitions as an active athlete. Before testing, athletes provided informed consent to participate in the study, which was approved by the Institutional Review Board of Behavioral and Social Sciences Research at National Taiwan University, approval number: 202004EM022.

### 2.2 Procedures

This study was randomized, cross-over, non-blinded design. Participants were first briefed on the experimental procedures by the researchers. Once they fully understood the purpose and methods of the study, the experiment began. Prior to the start of the experiment, the researchers fixed reflective markers to the participants to facilitate the capture of lower limb kinematic and kinetic parameters during the fencing lunge using a motion analysis system (Raptor-E Digital RealTime System, Motion Analysis Corporation, Santa Rosa, CA, USA) synchronized with an AMTI force plate (bp-600900, AMTI, Watertown, MA, USA). The infrared high-speed cameras were set to a capture frequency of 150 Hz, while the force plate was sampled at a frequency of 1,500 Hz.

The reflective markers were placed in the following locations (Modified Helen Hayes model): top head, front head, rear head, right medial scapula (offset), sacrum, and bilateral sides of acromion, lateral elbow, lateral wrist, anterior superior iliac spine (ASIS), mid-lateral thigh, medial knee epicondyle, lateral knee epicondyle, mid-lateral shank, medial ankle malleolus, lateral ankle malleolus, second metatarsal, and heel (Williams et al., 2019). In total, 29 markers were used, with the markers on the second metatarsal and heel attached to the athlete’s shoes. After the reflective markers were placed, the participants began their usual warm-up routine, which lasted approximately 5 min, before starting the formal test. The participants performed a long-lunge test in random order wearing court shoes (ASICS Upcourt 4, ASICS, Kobe-Shi, Hyogo-ken, Japan), fencing shoes (Nike Air Zoom Fencing Shoes, Nike, Washington County, OR, USA), or the same fencing shoes with a heel cup (Heelcare Cushion Cups 330., LP Support, Seattle, WA, USA) to compare the effects of the three types of footwear on the performance and lower limb loads of fencing athletes. The court shoes were made by synthetic fiber, artificial leather, and PVC sole (sole thickness 3.2 cm, heel-toe drop 2 cm, average weight 235 g). The fencing shoes were made by synthetic leather and polyester sole (sole thickness 2.7 cm, heel-toe drop 0.9 cm, weight around 275 g). The heel cup used in this study was made of silicone. (size 8*6*4 cm and weight 100 g). Only one force plate was available, so the test for each lower limb loads for each shoe condition were conducted separately. The test with only the front foot stepping on the force plate was performed three times, and the test with only the rear foot pushing off the force plate was performed three times. The average of the three tests for each foot represented the lower limb loads of the participant during the long lunge task. Rest time between each test was approximately 30 s.

During the long lunge task, the target was a humanoid figure standing at a height of 172 cm and width of 46 cm, placed at a distance 1.4 times the participant’s height in front of the participant’s front heel while in the lunge position. The figure was covered with a fencing suit to prevent the figure from being pierced by the sword. At the top of the figure’s head, a signal light and a 24 g accelerometer (Noraxon U.S.A. Inc., Scottsdale, AZ, USA) were set. The signal sampling frequency of both devices was set to 1,500 Hz, synchronized with the motion analysis system and the force plate. When the signal light was turned on, the participant lunged attack at the figure’s chest as quickly as possible, and the instant the figure was hit was detected by the accelerometer when the acceleration value in the hitting direction started to exceed 2 standard deviations from the noise level of the accelerometer in the absence of contact.

### 2.3 Data analysis

The Cortex 8 motion analysis software (Motion Analysis Corporation, Santa Rosa, CA, USA) was used for kinematic and kinetic analysis. The time between the signal light turning on and the moment the sword struck the target was defined as the attack time. Kinematic and kinetic data extraction included the maximum flexion and extension angles and angular velocities of the knee and ankle joints of the front foot and rear foot, as well as their maximum joint forces and moments, and the maximum GRF in the anterior-posterior, medial-lateral, and vertical axes. The LR of the front foot was calculated as the peak vertical GRF divided by the time to reach the peak vertical GRF from ground contact. The ground contact is defined as the moment when the vertical GRF starts to exceed 2 standard deviations from the noise level of the force plate in the absence of contact.

In addition, the distance between the front foot COP and the maximum forward position of the body COG at the same moment was calculated to represent the forward extension of the body. A shorter distance indicates greater stability of the front foot and more confidence to move the COG forward (Sato et al., 2022). The range of COP while standing on the force plate was also calculated for each foot to compare the control of the COP under different shoe conditions. The method for calculating the range of COP movement of the front foot involves subtracting the COP coordinates in the anterior-posterior or lateral-medial direction at the moment of ground contact from the maximum COP coordinates in the same direction. Similarly, for the rear foot, the range of COP movement in the anterior-posterior and lateral-medial directions is computed by subtracting the minimum COP coordinates after the signal light flashes from the maximum COP coordinates in the corresponding direction.

### 2.4 Statistical analysis

Statistical analysis was performed using SPSS Statistics software version 22 (SPSS Inc., Chicago, IL, USA). One-way ANOVA with repeated measures was used to compare the parameters among the three shoe conditions, and *post hoc* LSD tests were conducted to determine the differences between significant conditions at a significance level of α = 0.05.

## 3 Results

### 3.1 Attack time and kinematic parameters of lower limbs

All participants performed the lunge motion wearing three different types of shoes: court shoes, fencing shoes, and fencing shoes with a heel cup. The differences in the attack time and lower limb kinematic parameters were compared and presented in Table 1. The results showed that the attack time was significantly shorter when wearing fencing shoes (0.92 ± 0.05 s, *p* = 0.036) and fencing shoes with a heel cup (0.93 ± 0.07 s, *p* = 0.009), compared to when wearing court shoes (0.96 ± 0.06 s). There was no significant difference in the attack time when wearing fencing shoes with or without a heel cup. During the front foot stepping motion, there was no significant difference in the maximum movement angles and angular velocities of ankle and knee joints among the three shoe conditions. However, during the rear foot pushing motion, the maximum angular velocity of the ankle plantar flexion was significantly slower when wearing fencing shoes (326.2 ± 32.16°/sec, *p* = 0.033) and fencing shoes with a heel cup (316.93 ± 31.12°/sec, *p* = 0.008) than when wearing court shoes (369.29 ± 54.37°/sec). There was no significant difference in the maximum angular velocity of the ankle plantar flexion when wearing fencing shoes with or without a heel cup.

**TABLE 1:** Attack time and kinematic parameters of lower limbs.

	Fencing shoes with a heel cup (C1)	Fencing shoes (C2)	Court shoes (C3)	ANOVA (*p*-value)	LSD (*p*-value)		
					C1vs.C2	C1vs.C3	C2vs.C3
Attack time (sec)	0.93 ± 0.07	0.92 ± 0.05	0.96 ± 0.06	0.022*	0.461	0.009*	0.036*
**Front foot**							
Knee max. flexion angle (˚)	81.72 ± 8.13	86.46 ± 8.81	86.86 ± 13.33	0.217			
Knee max. extension velocity (˚/sec)	369.19 ± 93.26	374.41 ± 93.62	382.95 ± 99.83	0.387			
Knee max. flexion velocity (˚/sec)	271.53 ± 27.54	275.47 ± 30.69	272.15 ± 38.10	0.376			
Ankle max. dorsiflexion angle (˚)	19.30 ± 5.40	19.61 ± 4.11	17.29 ± 4.37	0.093			
Ankle max. plantarflexion angle (˚)	21.69 ± 9.54	22.43 ± 6.50	23.04 ± 10.14	0.342			
Ankle max. dorsiflexion velocity (˚/sec)	400.93 ± 112.15	384.50 ± 119.78	426.32 ± 135.53	0.140			
Ankle max. plantarflexion velocity (˚/sec)	604.79 ± 105.87	571.46 ± 62.92	563.18 ± 95.86	0.186			
**Rear foot**							
Knee max. flexion angle (˚)	50.34 ± 12.72	50.89 ± 11.17	49.13 ± 11.71	0.083			
Knee max. extension velocity (˚/sec)	245.23 ± 30.39	250.30 ± 21.11	240.74 ± 32.28	0.453			
Knee max. flexion velocity (˚/sec)	46.45 ± 22.18	50.15 ± 22.13	48.57 ± 15.65	0.792			
Ankle max. dorsiflexion angle (˚)	30.35 ± 6.89	29.59 ± 6.49	30.65 ± 5.82	0.205			
Ankle max. plantarflexion angle (˚)	40.39 ± 4.85	42.64 ± 6.72	40.84 ± 7.38	0.276			
Ankle max. dorsiflexion velocity (˚/sec)	131.12 ± 43.52	158.02 ± 76.82	152.92 ± 49.81	0.144			
Ankle max. plantarflexion velocity (˚/sec)	316.93 ± 31.12	326.2 ± 32.16	369.29 ± 54.37	0.013*	0.211	0.008*	0.033*

*: *p* < 0.05. The attack time was defined as the interval between the moment when the signal light turned on and the moment when the athlete hit the target.

### 3.2 Kinetic parameters of lower limbs

Differences in lower limb kinetics are summarized in Table 2. The study found that during the front foot stepping motion, the maximum posterior shear force of the knee joint (15.55 ± 7.63 vs. 10.54 ± 4.48 N/kg, *p* = 0.017), the maximum flexion moment of the knee joint (3.45 ± 1.75 vs. 2.48 ± 1.11 Nm/(kg·m), *p* = 0.040), and the maximum medial shear force of the ankle joint (5.24 ± 2.46 vs. 2.77 ± 1.94 N/kg, *p* = 0.001) were significantly greater when wearing fencing shoes compared to when wearing court shoes. The addition of a heel cup to fencing shoes resulted in a significant or near-significant reduction in these forces compared to not using a heel cup, but there was no significant difference compared to wearing court shoes. During the rear foot pushing motion, the maximum medial shear force of the ankle joint was highest when wearing fencing shoes (3.05 ± 1.73 N/kg, *p* = 0.007). This force decreased with the addition of a heel cup (2.44 ± 1.33 N/kg, *p* = 0.030) but remained greater than when wearing court shoes (1.79 ± 1.04 N/kg, *p* = 0.035).

**TABLE 2:** Kinetic parameters of lower limbs.

	Fencing shoes with a heel cup (C1)	Fencing shoes (C2)	Court shoes (C3)	ANOVA (*p*-value)	LSD (*p*-value)		
					C1vs.C2	C1vs.C3	C2vs.C3
**Front foot**							
Knee max. anterior force (N/kg)	4.81 ± 1.57	5.38 ± 0.82	5.03 ± 1.76	0.458			
Knee max. posterior force (N/kg)	12.31 ± 5.27	15.55 ± 7.63	10.54 ± 4.48	0.009*	0.051	0.121	0.017*
Knee max. vertical force (N/kg)	19.31 ± 3.25	18.75 ± 3.7	19.74 ± 4.01	0.872			
Knee max. flexion moment (Nm/(kg·m))	2.85 ± 1.28	3.45 ± 1.75	2.48 ± 1.11	0.028*	0.051	0.237	0.040*
Knee max. extension moment (Nm/(kg·m))	0.88 ± 0.26	0.87 ± 0.26	0.82 ± 0.23	0.262			
Ankle max. anterior force (N/kg)	10.28 ± 1.39	10.32 ± 1.34	9.37 ± 1.50	0.328			
Ankle max. posterior force (N/kg)	8.84 ± 3.64	10.61 ± 3.83	8.64 ± 3.21	0.112			
Ankle max. medial force (N/kg)	3.41 ± 2.13	5.24 ± 2.46	2.77 ± 1.94	<0.001*	<0.001*	0.348	0.001*
Ankle max. lateral force (N/kg)	0.68 ± 0.64	0.25 ± 0.08	0.83 ± 0.69	0.053			
Ankle max. vertical force (N/kg)	23.61 ± 4.88	25.09 ± 7.57	22.62 ± 5.12	0.397			
Ankle max. plantar flexion moment (Nm/(kg·m))	0.43 ± 0.11	0.47 ± 0.13	0.43 ± 0.13	0.309			
Ankle max. dorsiflexion moment (Nm/(kg·m))	0.42 ± 0.10	0.43 ± 0.10	0.41 ± 0.10	0.923			
Rear foot							
Knee max. anterior force (N/kg)	6.57 ± 2.00	6.57 ± 1.83	6.47 ± 2.04	0.894			
Knee max. posterior force (N/kg)	1.00 ± 0.57	1.12 ± 0.58	1.33 ± 0.75	0.117			
Knee max. vertical force (N/kg)	13.15 ± 2.22	12.67 ± 1.24	12.61 ± 1.40	0.372			
Knee max. flexion moment (Nm/(kg·m))	0.48 ± 0.14	0.50 ± 0.12	0.50 ± 0.16	0.313			
Knee max. extension moment (Nm/(kg·m))	0.87 ± 0.29	0.79 ± 0.26	0.80 ± 0.21	0.651			
Ankle max. anterior force (N/kg)	10.28 ± 1.39	10.32 ± 1.34	9.37 ± 1.50	0.328			
Ankle max. posterior force (N/kg)	8.84 ± 3.64	10.61 ± 3.83	8.64 ± 3.21	0.112			
Ankle max. medial force (N/kg)	2.44 ± 1.33	3.05 ± 1.73	1.79 ± 1.04	0.002*	0.030*	0.035*	0.007*
Ankle max. lateral force (N/kg)	2.26 ± 0.85	1.93 ± 0.88	2.55 ± 1.13	0.095			
Ankle max. vertical force (N/kg)	14.85 ± 2.08	14.43 ± 2.11	14.45 ± 2.51	0.640			
Ankle max. plantar flexion moment (Nm/(kg·m))	0.96 ± 0.18	0.96 ± 0.19	0.93 ± 0.16	0.579			
Ankle max. dorsiflexion moment (Nm/(kg·m))	0.03 ± 0.02	0.04 ± 0.03	0.06 ± 0.04	0.463			

*: *p* < 0.05.

### 3.3 Force plate parameters

The differences in GRF, forward extension of the body, COP displacement, and LR of the front foot during the lunge attack action were compared among the three shoe conditions. The results are presented in Table 3. The study found no significant differences in the distance between the front foot COP and the maximum forward position of the body COG at the same moment, indicating similar forward body extension performance across different shoe conditions. During front foot stepping and rear foot pushing, there were no significant differences in the maximum GRF and COP displacement range in the anterior-posterior and medial-lateral directions on the plantar surface among the three shoe conditions. The LR of the front foot exhibited significant differences among footwear conditions (*p* = 0.001). Post hoc analysis revealed that wearing fencing shoes resulted in a significantly higher LR compared to wearing court shoes (*p* = 0.002) or fencing shoes with a heel cup (*p* = 0.003). Furthermore, wearing fencing shoes with a heel cup demonstrated a higher LR compared to wearing court shoes (*p* = 0.010).

**TABLE 3:** Force plate parameters.

	Fencing shoes with a heel cup (C1)	Fencing shoes (C2)	Court shoes (C3)	ANOVA (*p*-value)	LSD (*p*-value)		
					C1vs.C2	C1vs.C3	C2vs.C3
COPx-COGx (mm)	435.19 ± 125.37	443.98 ± 105.36	380.19 ± 75.12	0.265			
**Front foot**							
Max. anterior GRF (N)	447.12 ± 61.85	456.84 ± 80.76	424.24 ± 75.44	0.269			
Max. medial GRF (N)	101.14 ± 49.11	95.73 ± 39.13	87.86 ± 43.63	0.556			
Max. lateral GRF (N)	51.54 ± 28.62	44.28 ± 25.24	40.1 ± 26.63	0.092			
Max. vertical GRF (N)	1,493.73 ± 287.29	1,579.53 ± 418.61	1,367.71 ± 247.97	0.176			
Anterior-posterior COP range (mm)	195.08 ± 74.96	202.57 ± 20.48	218.42 ± 38.01	0.454			
Medial-lateral COP range (mm)	45.63 ± 8.20	46.63 ± 13.27	37.05 ± 8.59	0.138			
Loading rate (BW/s)	320.03 ± 162.07	435.98 ± 253.45	208.45 ± 66.83	0.001*	0.003*	0.010*	0.002*
**Rear foot**							
Max. anterior GRF (N)	459.94 ± 80.83	429.17 ± 65.92	426.16 ± 84.08	0.721			
Max. lateral GRF (N)	59.69 ± 30.96	62.73 ± 35.53	54.80 ± 32.00	0.181			
Max. medial GRF (N)	48.83 ± 18.39	46.16 ± 18.53	46.52 ± 16.79	0.733			
Max. vertical GRF (N)	740.29 ± 116.93	744.8 ± 98.32	743.89 ± 116.94	0.911			
Anterior-posterior COP range (mm)	308.59 ± 57.57	289.54 ± 85.80	259.79 ± 91.40	0.635			
Medial-lateral COP range (mm)	118.37 ± 72.02	138.80 ± 67.65	134.59 ± 46.91	0.261			

COPx-COGx, represents the forward extension of body; COP, range is the range of COP, movement after front foot contact or during rear foot push off.

## 4 Discussion

The lunge attack in fencing is influenced by the force-time characteristics and coordination of the upper and lower extremities. The purpose of this study was to investigate the effects of specialized fencing shoes on attack time, lower limb kinematics, and kinetics during the lunge attack. Additionally, the study aimed to examine the potential benefits of using a cushioned heel cup in conjunction with the fencing shoes. The results revealed that wearing fencing shoes, with or without a heel cup, significantly reduced the attack time compared to court shoes. However, wearing fencing shoes led to increased joint loads in the lower limbs and a higher LR on the front foot. The use of a heel cup, on the other hand, helped attenuate these forces, effectively mitigating the impact.

Fencing is a high-intensity and combative sport that requires coordinated movements of the entire body. Therefore, the ability to perform technical hand actions and swift footwork is essential for fencers in various competitions and confrontations. The lunge attack in fencing, which involves the greatest range of motion and the longest displacement, is frequently executed during regular training and formal competitions (Aquili et al., 2013; Turner et al., 2014; Guan et al., 2018). It requires the fencer to take a large step forward with the front foot while pushing off with the rear leg. The results of this study demonstrated that attack times were effectively reduced when wearing fencing shoes, regardless of the use of a heel cup. Unlike court shoes that are primarily designed for indoor court sports with greater sole thickness and heel-toe drop, fencing shoes have a thinner outsole, providing better traction and making them more suitable for abrupt stops, forward and backward movements, and lateral shifts during fencing matches (Geil, 2002). They enable a more efficient transmission of the forward propulsive force generated by fencers to the ground, resulting in improved velocity (Geil, 2002). This phenomenon can be observed from the results of our study that fencers demonstrated a higher LR on their front foot during lunge attack when wearing fencing shoes.

The kinematic results showed that when wearing court shoes, the maximum angular velocity of ankle plantar flexion during the push-off phase in rear foot was greater compared to fencing shoes. This may be primarily due to the design of the inner edge of the court shoe sole, which protrudes more than that of fencing shoes, causing an impediment to smooth push-off during the stepping process. As a result, there is a rapid tilting phenomenon when the COP shifts to the edge, leading to faster ankle plantar flexion speed. A study comparing elite collegiate fencers and intermediate fencers (with training experience of 7.1 ± 1.2 years and 6.3 ± 2.4 years, respectively) did not find significant differences in the maximum movement angles and angular velocities of the lower limb joints during the lunge movement (Guan et al., 2018). The participants in the current study had an average training experience of 6.0 ± 3.0 years and were all experienced fencers. The lunge movement had been trained for a long time, resulting in a stable execution of the movement. In comparison with previous research, it was found that the joint movement angles and angular velocities of lower limbs during the lunge process were consistently stable among different individuals and when wearing different shoes, as long as the training experience and movement proficiency were sufficient (Guan et al., 2018). The recruited participants in this study had at least 2 years of training experience and were currently active fencers. Comparing the movement performance of the same individual wearing different shoes, there were minimal differences in the kinematics, indicating a high consistency in the execution of movements for each fencer.

The results of this study demonstrated that during the forward stepping motion in front foot, wearing fencing shoes resulted in greater posterior shear force and flexion moment at the knee joint, as well as medial shear force at the ankle joint. However, when combined with a heel cup, these increases in joint forces could be attenuated. A similar phenomenon was observed during the push-off motion in rear foot, where increased medial shear force at the ankle joint was observed. The primary reason for these findings is likely attributed to the design of the fencing shoe, specifically the curved shape of the heel position, which directly receives the initial and maximum impact forces. Additionally, the thinner sole design of fencing shoes, compared to the thicker and softer court shoes, contributes to less shock absorption (Geil, 2002), leading to greater force transmission to the ankle and knee joints. However, this design is intended to provide fencers with increased flexibility, smoothness, and multidirectional mobility (Geil, 2002). The recruited participants in this study were active and experienced fencers, including two Asian Games athletes and two youth national team athletes. Thus, wearing fencing shoes during the lunge movement allows for better execution of the technique, resulting in faster movement speeds, accompanied by increased forces experienced by the lower limb joints. A study by Guan et al. (2018) also indicated that elite fencers exhibit higher knee and hip joint moments compared to intermediate fencers, further highlighting the influence of movement speed on the forces experienced by the lower limb joints. A previous study comparing fencing shoes with court shoes have shown a 15.37% increase in plantar pressure when wearing fencing shoes during the lunge movement (Geil, 2002). However, this study did not investigate kinetics of the lower limb joints, providing limited understanding of the differences between shoes.

Some fencers often use a heel cup during regular competitions or training to reduce the impact on their heels, thus alleviating discomfort during the movements. This study is the first to investigate the effects of a heel cup specifically in the context of fencing. The heel cup utilized in this study was made of silicone with a softer center pad. They were designed to absorb excess pressure on the heel, providing cushioning and assisting in the regulation of weight distribution. The results of this study showed that, despite the soft material composition of the heel cup and its ability to absorb a certain amount of force, the use of the heel cup resulted in a reduction in ankle joint force and the LR of the front foot compared to wearing fencing shoes without a heel cup. Importantly, this reduction did not impact the generation of propulsive force during forward push-off and attack time. Furthermore, the overall speed of the lunge attack relies on the power and speed of the push-off from the rear foot, and the COP of the rear foot moves from the center foot to the forefoot. Therefore, the displacement of the COP during the push-off does not start from the heel (Chen et al., 2017). As such, the cushioning material located in the heel should have minimal impact on the generation of the rear-leg pushing force and the subsequent timing of the attack. Fencing emphasizes sudden movements and rapid stops, which impose significant force and impact on the feet. A well-designed fencing shoe needs to be able to provide efficient transmission of the forward propulsive force and have good shock absorption to reduce the impact on the heel during lunges.

This study has several limitations and potential implications for practical application. First, there were a variety in fencer’s levels and their training experience. Although all participants had at least 2 years of experience and were mostly national-level competitors, some individuals with longer training experience (9–11 years) were international-level adult or youth national team fencers. The difference in training experience and adaptation to different shoes may have influenced the experimental results. Additionally, the participants recruited for the study were females who generally have a lighter body weight and may experience less impact load on the shoe soles. It is important to consider that fencers with heavier body weights or male fencers using the same shoe models and heel cup as in this study may experience different cushioning effects. These differences in body weight should be carefully considered in practical applications. The fencers in this study were accustomed to wearing fencing shoes during their regular training and competitions, which may have influenced their control and familiarity with court shoes. Finally, the testing protocol in this study involved a single lunge movement. However, in actual competitions, fencers utilize a variety of complex techniques, including running, jumping, forward and backward movements, attacking, and defending. Future research should further investigate the effects of different shoe models and heel cup designs on other movements. This will offer coaches and fencers more comprehensive information for practical application.

## 5 Conclusion

The results of this study demonstrated that wearing fencing shoes significantly reduces the attack time and using a heel cup effectively reduces the forces in the lower limb joints. It is recommended that fencers wear fencing shoes during their regular training and official competitions. However, in practical application, variations exist among different models of fencing shoes and heel cup designs. Individuals should choose and adapt accordingly to achieve optimal results. Future research can further explore various factors, such as different shoe models, individual characteristics, and changing targets, to develop more personalized application guidelines.

## Data Availability

The raw data supporting the conclusion of this article will be made available by the authors, without undue reservation.

## References

[B1] Aquili A. Tancredi V. Triossi T. De Sanctis D. Padua E. DʼArcangelo G. (2013). Performance analysis in saber. J. Strength Cond. Res. 27 (3), 624–630. 10.1519/JSC.0b013e318257803f 23443217

[B2] Bottoms L. Greenhalgh A. Sinclair J. (2013). Kinematic determinants of weapon velocity during the fencing lunge in experienced épée fencers. Acta Bioeng. Biomech. 15 (4), 109–113. 10.5277/abb130414 24479483

[B3] Chen T. L. Wong D. W. Wang Y. Ren S. Yan F. Zhang M. (2017). Biomechanics of fencing sport: a scoping review. PloS one 12 (2), e0171578. 10.1371/journal.pone.0171578 28187164 PMC5302478

[B4] di Cagno A. Iuliano E. Buonsenso A. Giombini A. Di Martino G. Parisi A. (2020). Effects of accentuated eccentric training vs plyometric training on performance of young elite fencers. J. Sports Sci. Med. 19 (4), 703–713. 33239944 PMC7675629

[B5] Geil M. D. (2002). The role of footwear on kinematics and plantar foot pressure in fencing. J. Appl. Biomech. 18 (2), 155–162. 10.1123/jab.18.2.155

[B6] Gholipour M. Tabrizi A. Farahmand F. (2008). Kinematics analysis of lunge fencing using stereophotogrametry. World J. Sport Sci. 1 (1), 32–37.

[B7] Guan Y. Guo L. Wu N. Zhang L. Warburton D. E. R. (2018). Biomechanical insights into the determinants of speed in the fencing lunge. Eur. J. Sport Sci. 18 (2), 201–208. 10.1080/17461391.2017.1414886 29249174

[B8] Guilhem G. Giroux C. Couturier A. Chollet D. Rabita G. (2014). Mechanical and muscular coordination patterns during a high-level fencing assault. Med. Sci. Sports Exerc 46 (2), 341–350. 10.1249/MSS.0b013e3182a6401b 24441214

[B9] Gutiérrez-Dávila M. Rojas F. J. Caletti M. Antonio R. Navarro E. (2013). Effect of target change during the simple attack in fencing. J. Sports Sci. 31 (10), 1100–1107. 10.1080/02640414.2013.770908 23421933

[B10] Lin C.-Y. (2020). Human body dynamics detection of shock absorption and rebound ability of specialized athletic shoes. J. Adv. Comput. Intell. Intell. Inf. 24 (3), 293–298. 10.20965/jaciii.2020.p0293

[B11] Magnani C. Defrasne Ait-Said E. (2021). Geometrical analysis of motion schemes on fencing experts from competition videos. PloS one 16 (12), e0261888. 10.1371/journal.pone.0261888 34969042 PMC8717994

[B12] Perhamre S. Lundin F. Klassbo M. Norlin R. (2012). A heel cup improves the function of the heel pad in Sever's injury: effects on heel pad thickness, peak pressure and pain. Scand. J. Med. Sci. Sports 22 (4), 516–522. 10.1111/j.1600-0838.2010.01266.x 21410537

[B13] Reinschmidt C. Nigg B. M. (2000). Current issues in the design of running and court shoes. Sportverletz Sportschaden 14 (3), 72–81. 10.1055/s-2000-7866 11081243

[B14] Sato H. Nomura Y. Kamide K. (2022). Relationship between static balance and gait parameters in preschool children. Gait Posture 96, 143–148. 10.1016/j.gaitpost.2022.05.029 35660238

[B15] Thompson K. Chang G. Alaia M. Jazrawi L. Gonzalez-Lomas G. (2022). Lower extremity injuries in U.S. national fencing team members and U.S. fencing Olympians. Phys. Sportsmed. 50 (3), 212–217. 10.1080/00913847.2021.1895693 33625317

[B16] Trautmann C. Martinelli N. Rosenbaum D. (2011). Foot loading characteristics during three fencing-specific movements. J. Sports Sci. 29 (15), 1585–1592. 10.1080/02640414.2011.605458 22077403

[B17] Turner A. Bishop C. Chavda S. Edwards M. Brazier J. Kilduff L. P. (2016). Physical characteristics underpinning lunging and change of direction speed in fencing. J. Strength Cond. Res. 30 (8), 2235–2241. 10.1519/jsc.0000000000001320 26808849

[B18] Turner A. James N. Dimitriou L. Greenhalgh A. Moody J. Fulcher D. (2014). Determinants of olympic fencing performance and implications for strength and conditioning training. J. Strength Cond. Res. 28 (10), 3001–3011. 10.1519/jsc.0000000000000478 24714533

[B19] Williams C. C. Gdovin J. R. Wilson S. J. Cazas-Moreno V. L. Eason J. D. Hoke E. L. (2019). The effects of various weighted implements on baseball swing kinematics in collegiate baseball players. J. Strength Cond. Res. 33 (5), 1347–1353. 10.1519/JSC.0000000000002020 29019867

[B20] Williams L. R. Walmsley A. (2000). Response timing and muscular coordination in fencing: a comparison of elite and novice fencers. J. Sci. Med. Sport 3 (4), 460–475. 10.1016/s1440-2440(00)80011-0 11235010

